# P02.79. Study on the basic tongue diagnosis indicators for pattern identifications in stroke using a decision tree method

**DOI:** 10.1186/1472-6882-12-S1-P135

**Published:** 2012-06-12

**Authors:** J Lee, M Ko, T Park, B Kang, T Moon, M Lee

**Affiliations:** 1 Korean Institute of Oriental Medicine, Daejeon, Republic of Korea

## Purpose

The purpose of this study is to select the major tongue diagnosis indicators and evaluate their significance in discriminating the subtypes of Pattern Identification (PI) from stroke patients.

## Methods

Decision tree analysis was carried out using clinical data collected from 1502 stroke patients with same subtypes diagnosed identically by two experts with more than 3 years of clinical experience. Among 9 tongue indicators, 6 major tongue indicators (red tongue, pale tongue, yellow fur, white fur, thick fur, teeth-marked tongue) were selected by decision tree analysis. Each PI has a specific combination of tongue indicators which related 6 major tongue indicators.

## Results

It is suggested that 6 tongue indicators can be used for discrimination of PI in stroke patients, though the combination studies between these tongue indicators and the other PI indicators are left for further study.

## Conclusion

We could conclude that tongue diagnosis can play a significant role in a differential diagnosis of PI. Namely it can increase the accuracy of diagnosis and simplify the complex process of PI to check important tongue indicators. It is necessary to re-evaluate other indicators in PI by proper ways, and this study is helpful to objectify and be scientific in traditional Korean medicine.

